# The Effect of Demonstrator Social Rank on the Attentiveness and Motivation of Pigs to Positively Interact with Their Human Caretakers

**DOI:** 10.3390/ani11072140

**Published:** 2021-07-20

**Authors:** Daniela Luna, Catalina González, Christopher J. Byrd, Rocío Palomo, Elizabeth Huenul, Jaime Figueroa

**Affiliations:** 1 Departamento de Ciencias Animales, Facultad de Agronomía e Ingeniería Forestal, Pontificia Universidad Católica de Chile, Santiago 7820436, Chile; dlunavet@gmail.com (D.L.); cata2403@gmail.com (C.G.); ekhuenul@uc.cl (E.H.); 2 Deparment of Animal Sciences, North Dakota State University, NDSU Dept. 7630, P.O. Box 6050, Fargo, ND 58108-6050, USA; christopher.byrd@ndsu.edu; 3 Departamento de Fomento de la Producción Animal, Facultad de Ciencias Veterinarias y Pecuarias, Universidad de Chile, Santa Rosa 11735, La Pintana, Santiago 8820000, Chile; rocio.palomo@ug.uchile.cl; 4 Instituto de Ciencias Agroalimentarias, Animales y Ambientales—ICA3, Universidad de O’Higgins, San Fernando 3070000, Chile

**Keywords:** attentional bias, human–pig relationship, pig welfare, social learning, social rank, social relationships, observational learning

## Abstract

**Simple Summary:**

Motivation to express a socially learned behavior can be inhibited or facilitated by the presence of socially dominant individuals, who often attract greater attention from their conspecifics. This study investigated whether experienced pigs (termed “demonstrators”) of higher rank attract greater attention from their pen mates when they are observed from behind an acrylic panel while being gently handled by the stockperson. We also investigated whether the presence of a demonstrator pig of different social rank, who previously established a positive relationship with the stockperson in presence of conspecifics, affects the motivation and behaviors of their pen mates to positively interact with humans. Our results show that during the gentle handling of the demonstrator, there was an overall preference for observer pigs to visually attend to the dominant demonstrators more than the low-ranking demonstrators. Furthermore, our study shows that the proximity of a dominant demonstrator pig interfered with the motivation of their pen mates to positively interact with the stockperson, whereas the presence of a subordinate demonstrator facilitated the expression of a greater affinity toward the human, resulting in longer physical contact, and a higher percentage of accepted strokes. These findings reveal that social dynamics and dominance rank have a strong effect on the attentional state and the facilitation and inhibition of social behaviors in domestic pigs.

**Abstract:**

In this study, we addressed the social attentiveness, as well as the phenomenon of social facilitation and inhibition in the context of a positive human–pig relationship. Specifically, we investigated whether the social rank of an experienced pig (termed “demonstrator”) has an effect on the attentiveness of the remaining pen mates (N = 40) when they observe the demonstrator being gently handled by a stockperson from behind an acrylic panel. We found that pigs preferentially attended to dominant demonstrators rather than subordinate demonstrators during their gentle handling sessions with the stockperson. Additionally, we also examined whether the presence of a demonstrator pig of different social rank, who previously established a positive relationship with the stockperson in presence of conspecifics, affects the behavior and motivation of their pen mates to positively interact with the stockperson. To test for the effect of the presence and demonstrator’s social rank on pen mate interactions with the stockperson, we evaluated the behavior of domestic pigs (N = 65) toward the stockperson using a human-approach test in their home-pen. Pigs showed a decrease in their motivation to positively interact with the stockperson when a socially dominant demonstrator was present, behaving similarly to animals receiving minimal human contact (control group). Overall, they exhibited a greater latency to physical contact, a lower acceptance of stroking, and spent more time looking at the stockperson compared to pigs exposed to subordinate demonstrators. Taken together, these findings expand our current understanding of pigs’ cognition and social behavior, and the nature of social attention bias in farm animals. Our findings indicate that positive handling of previously selected subordinate demonstrators seems to be the best strategy to reduce the level of fear in large groups of pigs.

## 1. Introduction

The human–animal relationship (HAR) is an important part of management in modern livestock production and an essential component of animal welfare assessment protocols in commercial swine production [1]. This becomes even more evident now that the Five Domains Model of animal welfare has been recently updated to include human–animal interactions (HAI) in its assessment of animal welfare [2]. The nature of interactions (positive, negative, or neutral) between the stockperson and their animals is an important factor in determining the degree of animals’ fear or affinity toward humans and, consequently, the quality of the HAR [3]. The quality of the HAR has a large effect on welfare, productivity, and ease of handling the animals [4].

Pigs are social animals that naturally seek interaction with humans and can establish a positive relationship with the stockperson after being regularly exposed to rewarding stimuli, such as stroking [5,6], food rewards [7], and/or the use of a soft gentle voice [8]. However, pigs often experience negative interactions (e.g., handling, medical intervention, transport) with their caregiver during most of their production cycle [9]. In addition, the large number of animals housed in intensive production systems limits the stockperson’s ability to provide regular positive contact, making it difficult to establish a positive human–pig relationship [6]. In consequence, it is a challenge to identify strategies that promote the development of positive stockperson–pig relationships.

Recent research has revealed that domestic animals can acquire a positive perception of humans through the observation of an experienced conspecific (termed “demonstrator”), while she/he is being gently handled by the human, a phenomenon attributed to observational social learning (guinea pigs: [10]; horses: [11]; cows: [12]; pigs: [6]. Social learning refers to the ability of an animal to acquire information from the social environment by observing or interacting with a more experienced individual [13]. For instance, Luna et al. [6] found that individuals who had observed a demonstrator pig being gently handled by a stockperson for five weeks, showed lower physiological stress levels, and greater affinity behaviors toward the stockperson in an open-field arena test, in comparison to those animals that received minimal human contact [6]. Social learning can be modulated by some intrinsic traits of the demonstrator individual, such as age [14], sex [15], prestige [16,17], and social rank [18]. Regarding social rank in the learning context, several studies show that animals may prefer to use the information provided by high-ranking individuals, as observed in laying hens (*Gallus gallus domesticus*) [19,20], horses (*Equus caballus*) [21], dogs (*Canis lupus familiaris*) [22], and chimpanzees (*Pan troglodytes*) [17,23]. For instance, Nicol and Pope [19] reported that that social learning of the keypeck response to obtain food was greater in laying hens who had observed socially dominant demonstrators in comparison to hens that had observed subordinate demonstrators. In Nicol and Pope’s study, when the observer hens were individually tested, the most correct keypecks were performed by the hens that previously had seen socially dominant demonstrators perform the task. Furthermore, during the demonstration sessions, the dominant demonstrator hens attracted the visual attention of a greater number of observer hens compared to subordinate demonstrators [19]. Accordingly, social transfer of information, as well the preferential visual attentiveness, seems to depend on the social relationship between observer and demonstrator, being the high-ranking individuals apparently more influential and visually more attractive demonstrators than low-ranking individuals [18,24].

Nevertheless, despite the wide evidence showing a dominance-based bias in social learning, some inconsistencies have been reported [25,26], suggesting differences between species. Luna et al. [6], found no differences in information acquisition about a positive perception of the human between pigs who previously observed socially dominant and subordinate individuals being gently handled by the stockperson. Pigs exposed to demonstrators showed greater affiliative behaviors toward the stockperson when they were individually tested outside their social group, regardless of whether they observed a dominant or subordinate demonstrator receiving gentle handling [6]. It should be noted, however, that information and/or behaviors that have been socially learned by an individually tested “observer” might not be expressed if animals are evaluated in their natural social environment (i.e., within their social group) [27]. It has been described that the presence of high-ranking or trained demonstrators may facilitate [28] but also inhibit the expression of social learning in their conspecifics [19,29]. This is commonly referred to as social facilitation and social inhibition, respectively [29]. In chimpanzees, for instance, the proximity of a dominant animal inhibited their subordinate conspecifics from performing a food acquisition task, in comparison to when they were tested in absence of the dominant [30]. Similar findings were reported in small flocks of laying hens during a keypeck test to obtained food. In a second experiment, Nicol and Pope [19] found that the removal of the Perspex screen separating demonstrator and observers decreased social learning in the observer hens, as they did not acquire the keypeck response in presence of the demonstrator. This was attributed to the fact that demonstrator hens threatened and displaced the observer animals to defend the key area [19]. Thus, group members’ motivation state to express their learning might differ according to whether experienced individuals, especially those high-ranking, are present or not [31]. Under commercial conditions, pigs are maintained in social groups, where members establish a social hierarchy and are in continuous physical contact with each other. In addition, pigs are commonly handled by the stockperson in a familiar physical and social environment. Consequently, more studies are required to further understand the influence of social hierarchy on social learning when the animals are confronted by a stockperson within their social group.

In this study, we investigated whether the social rank of the demonstrator pigs influences the social attentiveness they receive from their conspecifics during the demonstration sessions of positive interaction with the stockperson. We hypothesized that dominant demonstrator pigs attract greater visual attentiveness from their conspecifics, compared to subordinate demonstrators. Additionally, we address the phenomenon of social facilitation and inhibition in the context of positive human–pig relationships. Specifically, we aimed to examine whether the social rank of a conspecific demonstrator affects the motivation and behavior of their pen mates to positively interact with the stockperson. We hypothesized that the presence of high-ranking demonstrators interferes with the motivation of their groupmates to positively interact with the stockperson.

## 2. Materials and Methods

The experiments were performed at the weanling unit of the swine experimentation facility at the Pontificia Universidad Católica de Chile (PUC; Pirqué, Metropolitan Region, Chile). The Ethical Committee on Animal Experimentation of the PUC approved all procedures involving pigs (no. 180928004).

### 2.1. Animals and Housing

A total of 75 castrated male (*n* = 34) and female (*n* = 41) piglets (PIC Genetics) were weaned at 21 days of age and immediately transported from a commercial pig farm to the experimental pig facilities belonging to the PUC (day 1; Table 1). On arrival, the pigs were weighed (5.6 ± 0.2 kg) and randomly allocated to 15 nursery pens in groups of five pigs considering similar weights between pens (*p* > 0.05). Afterward, pigs were individually identified by using numbered plastic ear tags of different colors. The experimental swine facility consisted of 15 nursery pens of with solid walls (1.80 m × 1.28 m × 0.7 m) on a fully slatted floor. Inside each pen, pigs had access to a dry pellet feeder with space for three animals, and an independent nipple drinker next to the feeder. The nursery room was provided with a controlled environment temperature (27.3 ± 2.70 °C) and automatically forced ventilation. Pigs were fed with a standard commercial diet (Champion S.A, Santiago, Chile) according to the nutritional requirements set by the National Research Council [32]. Commercial feed and water were provided ad libitum for the entire experimental period. Animal health status was evaluated by visual inspection once a day. All pigs underwent a period of acclimation to the facilities and management conditions during the first post-weaning week (Table 1).

### 2.2. Procedures

#### 2.2.1. Determination of Social Dominance Order

Immediately after mixing and housing the pigs, the agonistic interactions in 10 randomly selected pens were registered by continuous video recording during day 1 and day 2 post-weaning (48 h; Table 1). To identify the pigs and facilitate the behavioral observations, they were previously marked with a number on their back using a non-permanent marker pen. Nine hours of focal continuous sampling during the first two days post-weaning (day 1: 1:00 to 10:00 p.m.; day 2: 9:00 a.m. to 6:00 p.m.) were used for the analysis aimed at establishing the social dominance order.

One video camera (IR Outdoor Cameras 700tvl 1/3 CMOS Sony, SENKO SA, Santiago, Chile) was placed on the ceiling of each pen to record pigs’ agonistic behaviors. Afterward, agonistic behaviors video collection was analyzed by 10 observers previously trained in the detection and identification of aggressive behaviors in pigs (days 3–5). For each agonistic interaction, the duration of the encounter, the aggressor, the receiver, the winner and the loser, and the inconclusive fights were registered by observers. Subsequently, a dominance index (DI) was calculated for each animal who was involved in agonistic interaction, based on the study performed by Stukenborg et al. [33]. The DI is ranked from −1 to +1 and was used to determine the most subordinate (the closest to −1) and the most dominant (the closest to +1) animal inside each pen, who then acted as a demonstrator (for more details see Luna et al. [6]). Behavioral observations were analyzed using the Behavioral Observation Research Interactive Software (BORIS version 7.8) [34].

#### 2.2.2. Treatments

During the second week post-weaning (Table 1), each pen was randomly allocated to one of the three treatments (5 pens/treatment; 5 pigs/pen) as follows:

Control Group (CG, *n* = 5 pens): all pigs in the pen received minimal human contact corresponding to daily routine practices for managing pigs under commercial conditions (i.e., feeding, cleaning, and health evaluation).

Dominant Demonstrator Group (DDG, *n* = 5 pens): four pigs (“observer pigs”) within the pen were exposed to their socially dominant “demonstrator pig” through a transparent acrylic panel while it was subjected to gentle human handling, according to a standardized procedure [6] (see gentle handling procedure section below).

Subordinate Demonstrator Group (SDG, *n* = 5 pens): four pigs (“observer pigs”) within the pen were exposed to their socially subordinate “demonstrator pig” under the same conditions described above for the DDG pigs.

Both treatment application and routine practices were conducted by two women stockpeople who wore overalls of different colors during the entire experimental period to facilitate human discrimination by pigs. One stockperson was in charge of the CG and wore green overalls and white boots, while the other one was responsible for the DDG and SDG animals and wore blue overalls and white boots. During treatment application, the stockpeople did not physically touch the pigs (except the demonstrator pig; see gentle handling procedure section below) unless it was absolutely necessary. The routine practices, such as fill feeders, pen cleaning, and health evaluations were performed outside the pens.

##### Gentle Handling Procedure

Briefly, each demonstrator pig was subjected to a 10 min gentle handling (GH) session twice daily (Monday–Friday between 9:00 and 11:00 a.m. and 2:00–4:00 p.m.) for a period of 5 weeks (Table 1). The GH session was standardized as follows (for more details see Luna et al. [6]): (1) 10 min before the start of the GH session, the pen was temporarily partitioned into two areas (demonstrator pig handling area and observer pig area) through the installation of a transparent acrylic panel with circular perforations (5 cm diameter) homogeneously distributed. (2) Afterward, the stockperson entered the pen, calmly caught the demonstrator pig, and proceeded to release it on the floor of the handling area. (3) Then, the stockperson entered the handling area, sat down on the floor, and remained motionless for 3 s. (4) Afterward, the stockperson reached out her hand toward the demonstrator pig. If the pig did not move away, then the stockperson attempted to stroke them gently with the palm of her hand, from head to back, at a rate of 1 stroke every 2 s for 7 min. (5) Over the subsequent 3 min, the stockperson offered the demonstrator pig a 16% sucrose reward solution while stroking it. In addition to gentle tactile contact, the stockperson spoke to the demonstrator pig using a soft voice during the GH session. (6) Once the GH was finished, the stockperson picked up the demonstrator pig and returned it to the observer pigs’ area with their pen mates, and proceeded to leave the pen. (7) Finally, 10 min after returning the demonstrator pig, the panel and the drinker bowl with the sucrose solution were removed.

#### 2.2.3. Behavioral Tests and Measurements

##### Behavioral Observations

The behavioral reactions of the observer pigs during/after the GH demonstration session, as well their behaviors toward the stockperson in the home pen at the end of the treatment period were recorded using video cameras (IR Outdoor Cameras 700tvl 1/3 CMOS Sony, SENKO SA, Santiago, Chile) fixed on the ceiling of each rearing pen. All animals were individually observed in their home pen by continuous focal sampling. The behavioral observations were analyzed using BORIS Software [34].

##### Behavior of the Observer Pigs during and after the Gentle Handling Sessions

The behavioral reactions of observer pigs from DDG and SDG toward the demonstrator–stockperson interactions (DSI) during the GH sessions were evaluated. The assessments were performed during the first GH session of each day (Monday through Friday; 9:00–11:00 a.m.) during the first (days 8–12; Table 1) and second (days 15–19; Table 1) weeks of GH sessions with the demonstrator pigs. Additionally, the behavior of observer pigs toward the demonstrator pig immediately after he/she had returned to their social group post-GH session was evaluated for 3 min. Behavioral observations evaluated during and after the GH sessions are described in Table 2.

##### Behavior of the Observer Pigs toward the Stockperson in the Home-Pen Test

Protocols, tests, and behavioral observations used in the present study were adapted from previous investigations that addressed the study of the human–pig relationship and interaction [5,6,35].

The behavioral reaction of pigs toward the stockperson in a familiar environment (home pen) with the demonstrator pig and pen mates present was evaluated during days 41 and 42 (Table 1). The test lasted 5 min and consisted of three phases: (1) the stockperson quietly entered the home pen and stood motionless for 1 min (Phase 1—Stockperson standing stationary: 0–1 min). (2) Then, the stockperson walked slowly toward the opposite wall of the pen, stopped for 1 s and then returned to the wall of the entrance of the pen, where she proceeded to sit motionless on the floor at the middle of the wall without interacting with the animals for 2 min (Phase 2—Stockperson sitting stationary: 1–3 min). (3) After Phase 2, and for the next 2 min, the stockperson extended her hands toward the pigs in order to stroke each pig randomly. If a pig accepted being touched, the stockperson softly stroked it along the body from head to back (1 stroke/2 s) with the palm of her hand (Phase 3—Stockperson moving: 3–5 min). Finally, after Phase 3, the stockperson slowly stood up and proceeded to leave the pen. Behavioral observations evaluated during the home-pen test are described in Table 3.

### 2.3. Statistics

Data analysis was performed using the SPSS version 22.0 (IBM Corp.; Armonk, NY, USA). For all analyses, the experimental unit was the pen, and the observational unit was the animal. Prior to analysis, the normality and homoscedasticity of the dataset were analyzed using the Shapiro–Wilk test and Levene test, respectively.

#### 2.3.1. During and after Gentle Handling Sessions

For behavioral measures evaluated during and after the GH sessions, differences between groups (DDG and SDG) were analyzed by week (week 1 and week 2) using repeated mixed ANOVA. The model included the treatment (DDG and SDG), sex (male and female), the day of the test (repeated variable), and their interactions as fixed effects. The pig identity and the pen were included as a random effect in all models. The interactions that had no significant influence in the models (sex × treatment; day × sex) were removed and the data set reanalyzed. For all models, post hoc comparisons after mixed ANOVA were run using least significant differences (LSD) [36]. When the residuals of the dataset did not follow a normal distribution, data were transformed to meet assumptions of statistical tests: behavioral data expressed as a percentage were subjected to angular transformation, while data expressed as total number of occurrences (frequencies) were square-root-transformed to obtain normally distributed residuals, according to recommendations of Martin and Bateson [37]. Results are presented as estimated marginal means (E.M.M.) and standard errors means (S.E.M.) of non-transformed data for the repeated mixed ANOVA. Additional data are reported using descriptive statistics (means ± S.E., percentage). For all analyses, a statistically significant difference between comparisons was defined as *p* < 0.05. A trend in the data was defined as 0.05 < *p* < 0.1.

#### 2.3.2. Home-Pen Test

Behavioral data obtained during the home-pen test were analyzed by phase. The data of demonstrators were removed from analysis to not overestimate the results. For behavioral measures, data were analyzed using mixed ANOVA. Treatment (DDG, SDG, and CG), sex (male and female), and their interaction were included as fixed effects, whereas the pen nested within treatment was included as a random effect in all models. When the sex and the interaction between treatment and sex had no significant influence in the models, these were removed, and the data set was reanalyzed. For all models, post hoc comparisons after mixed ANOVA were run using LSD. When the residuals of the dataset did not follow a normal distribution, data were transformed to meet assumptions of statistical tests (behavioral data expressed as a percentage were angular-transformed to obtain normally distributed residuals; [37]). When the criteria for parametric statistics using transformed data could not be met, data were analyzed using the non-parametric Kruskal–Wallis test with the post hoc Dunn–Bonferroni method. Results are presented as estimated marginal means (E.M.M.) and standard error means (S.E.M.) of non-transformed data for the mixed ANOVA and as medians and interquartile ranges (25th and 75th percentiles) for the non-parametric Kruskal–Wallis test. Additional data are reported using descriptive statistics (means ± S.E., percentage).

The variable of latency to first physical contact was analyzed using the non-parametric Kaplan–Meier survival method [38]. A log-rank significance test was performed to test whether latencies differed statistically between treatments. The results are presented as means and standard errors of the latencies.

Additionally, a Pearson’s chi-square test was conducted to determine the effect of treatment on the number of animals that performed the behavior of climbing on the stockperson (Phase 2 and Phase 3). Post-hoc comparisons after the chi-square test were run using the adjusted standardized residuals with Bonferroni adjusted *p*-value [39].

For all analyses, a statistically significant difference between comparisons was defined as *p* < 0.05. A trend in the data was defined as 0.05 < *p* < 0.1.

## 3. Results

### 3.1. Behavioral Reaction of Observer Pigs during and after the Gentle Handling Sessions

#### 3.1.1. First Week of Gentle Handling Sessions

For all behavioral measures, the values and the effects of treatment are summarized in Table 4. During the first week of gentle handling, observer pigs from DDG tended to look at the demonstrator-stockperson interaction (F1/35.74 = 3.320, *p* = 0.077) and to contact the panel (F1/36.30 = 3.693, *p* = 0.071) for a longer duration than observer pigs from SDG. However, no differences were found between the treatment groups regarding the frequencies of looking at DSI (F1/35.45 = 0.175, *p* = 0.678), contacting the panel (F1/34.64 = 0.272, *p* = 0.484) and climbing on the panel during week 1 (F1/40.05 = 0.524, *p* = 0.473).

**Figure 1:**
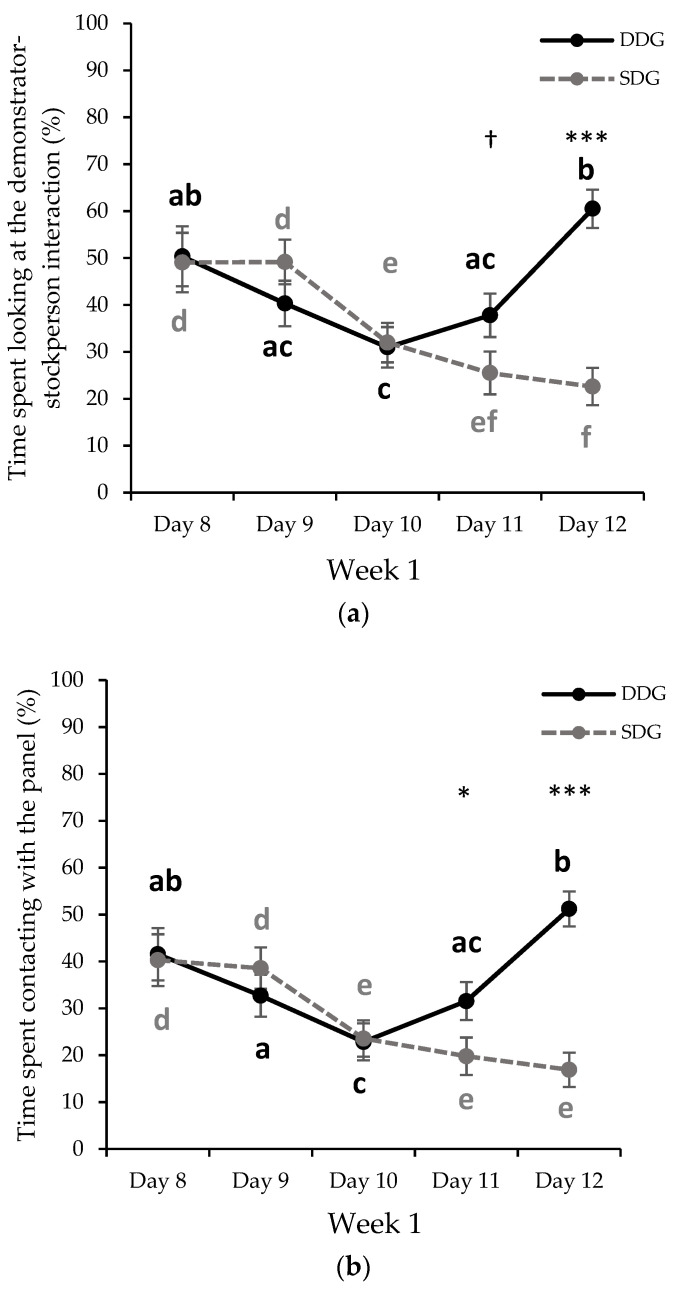
Change in the percentage mean (±S.E.M) of: (**a**) the time spent looking at the demonstrator-stockperson interaction and (**b**) the time of contact with the panel for DDG (*n* = 20) and SDG (*n* = 20) pigs during the gentle handling sessions during week 1 (days 8–12 of the experimental procedure). DDG = dominant demonstrator group (black); SDG = subordinate demonstrator group (light grey). Treatment effect by day: † 0.05 < *p* < 0.1; * *p* < 0.05; *** *p* < 0.001. Day effect within a treatment: values with different letter differ significantly (*p* < 0.05); a, b, c (black) correspond to DDG treatment, and d, e, f (light grey) to SDG treatment.

Regarding the effect of the GH session day (independent of the treatment) the pigs spent more time looking at the DSI (F4/66.10 = 5.545, *p* < 0.001) and contacting the panel (F4/64.56 = 6.126, *p* < 0.001) during the first (day 8, time looking: 49.70% ± 4.48; time in contact: 40.91 ± 3.92), second (day 9, time looking: 44.73% ± 3.38; time in contact: 35.63 ± 3.13), and the fifth day of GH session (day 12, time looking: 41.54% ± 2.83; time in contact: 34.05 ± 2.60). No differences between these days were observed (*p* > 0.10). However, it is worth highlighting that the greater time spent looking at the DSI and contacting the panel during the fifth day was mainly attributed to DDG pigs (Figure 1a,b). In contrast, the time that pigs spent looking at the DSI and contacting the panel showed a significant decrease during the third (day 10, time looking: 31.47% ± 3.01; time in contact: 23.21 ± 2.75) and fourth day (day 11, time looking: 34.64% ± 3.24; time in contact: 25.67 ± 2.83), observing no differences between both days (*p* > 0.10). Regarding the frequency of looking at the DSI (F4/56.75 = 4.401, *p* = 0.004), animals looked at the DSI more frequently during the first (day 8, 11.17 ± 1.05), second (day 9, 9.91 ± 0.61), and fifth day (day 12, 9.75 ± 0.86) compared to the third (7.65 ± 0.62) and fourth day (7.55 ± 0.66). No differences between the first, second, and fifth day were observed (*p* > 0.10). Regarding the frequency of contact with the panel (F4/39.18 = 4.765, *p* = 0.035), animals touched the panel more frequently only during the first (14.85 ± 1.32) and second day (13.91 ± 9.12). No additional differences between the remaining days were observed (*p* > 0.10). There was no effect of GH session day on the frequency of climbing on the panel (F4/58.10 = 1.768, *p* = 1.48).

There was an interaction between treatment and GH session day for the time that pigs spent looking at the DSI (F4/60.79 = 14.602, *p* < 0.001; Figure 1a) and the time of contact with the panel (F4/64.56 = 13.326, *p* < 0.001; Figure 1b). The observer pigs from the DDG spent more time looking at the DSI during the fifth day (day 12; DDG: 60.49% ± 4.07; SDG: 22.59% ± 3.98) of week 1, compared to pigs from SDG (*p* < 0.001; Figure 1a). In addition, a tendency was found during the fourth day (day 11), where the pigs from DDG tended to look at the DSI for a longer duration (DDG: 37.79 % ± 4.64; SDG: 25.51% ± 4.56) than pigs from SDG (*p* = 0.066) (Figure 1a). Regarding the time of contact with the panel, the pigs from DDG spent more time in contact during the fourth (day 11; DDG: 31.55% ± 4.07; SDG: 19.79% ± 3.99; *p* = 0.045) and fifth day of week 1 (day 12; DDG: 51.21% 3.74; SDG: 16.89% ± 3.65; *p* < 0.001) compared to pigs from SDG, while no differences were found during the other days (*p* > 0.10; Figure 1b). There was no interaction between treatment and GH session day for frequency of looking at the DSI (F4/54.07 = 1.446; *p* = 0.231), frequency of contact with the panel (F4/55.71 = 1.696; *p* = 0.164), and frequency of climbing on the panel (F4/58.10 = 0.253; *p* = 0.906).

After the GH sessions, no differences were found between the treatment groups regarding the time of snout–snout contact (F1/35.72 = 1.963, *p* = 0.170) and the frequency of snout–snout contact (F1/34.50 = 1.021, *p* = 0.319) with the demonstrator pig. There was no effect of GH session day on the time of snout–snout contact (F4/59.37 = 1.265; *p* = 0.294), the frequency of snout–snout contact (F4/58.96 = 1.291; *p* = 0.284), or interaction between treatment and GH session day for the preceding variables (*p >* 0.10).

No effects of observer pig sex were found for the behavioral measures during and after the GH session during week 1 (*p* > 0.10).

#### 3.1.2. Second Week of Gentle Handling Sessions

For all the behavioral measures, the values and the effects of treatment are summarized in Table 4. During the second week of GH, observer pigs from DDG spent more time looking at the demonstrator-stockperson interaction (F1/41.28 = 48.986, *p* < 0.001) and contacting the panel (F1/37.29 = 21.878, *p* < 0.001) than observer pigs from SDG. In addition, DDG observer pigs looked at the DSI (F1/46.33 = 5.916, *p* = 0.019) and touched the panel (F1/39.18 = 4.765, *p* = 0.035) more frequently than SDG pigs. No differences were found between the treatment groups regarding the frequency of climbing on the panel (F1/53.90 = 0.002, *p* = 0.961). Moreover, there was no significant effect of GH session day on the observed variables during week 2 (*p* > 0.10).

**Figure 2:**
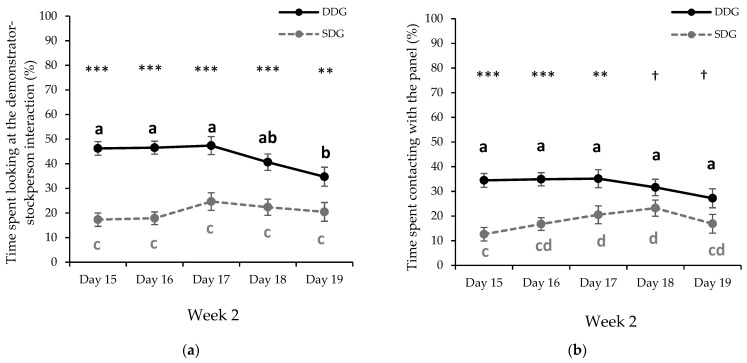
Change in the percentage mean (±S.E.M) of: (**a**) the time spent looking at the demonstrator-stockperson interaction and (**b**) the time of contact with the panel for DDG (*n* = 20) and SDG (*n* = 20) pigs during the gentle handling sessions at week 2 (days 15–19 of the experimental procedure). DDG = dominant demonstrator group (black); SDG = subordinate demonstrator group (light grey). Treatment effect by Day: † 0.05 < *p* < 0.1; ** *p* < 0.01; *** *p* < 0.001. Day effect within a treatment: values with different letter differ significantly (*p* < 0.05); a, b (black) correspond to DDG treatment, and c, d (light grey) to SDG treatment.

A significant interaction between treatment and GH session day for time spent looking at the DSI (F4/52.85 = 2.99, *p* = 0.027; Figure 2a) and the time of contact with the panel (F4/50.36 = 2.19, *p* = 0.043; Figure 2b) was found. The DDG observer pigs spent more time looking at the DSI than SDG pigs during each GH session day during week 2 (*p* < 0.001; Figure 2a). Regarding the time of contact with the panel, observer pigs from DDG spent more time contacting the panel than SDG pigs during the first three GH session days during week 2 (*p* < 0.01; Figure 2b). There was no interaction between the treatment and GH session day for frequency of looking at the DSI (F4/57.49 = 0.565; *p* = 0.689), frequency of contact with the panel (F4/56.42 = 0.359; *p* = 0.837), and frequency of climbing on the panel (F4/48.57 = 0.516; *p* = 0.725).

After the GH sessions, no differences were found between treatment groups regarding the time (F1/34.26= 0.096, *p* = 0.759) and frequency of snout–snout contact (F1/34.01= 0.026, *p* = 0.874) with the demonstrator pig. There was no effect of GH session day on the time F4/52.73 = 1.173; *p* = 0.330) or the frequency of snout–snout contact (F4/54.83 = 1.435; *p* = 0.235). No interaction between treatment and GH session day was observed for the preceding variables (*p* > 0.10).

### 3.2. Behavior toward the Stockperson in the Home-Pen Test

**Figure 3:**
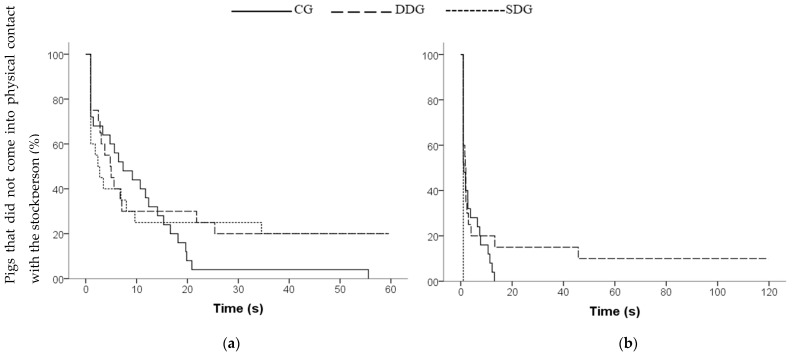
Survivor curve of pigs’ latencies to first contact with the stockperson during: (**a**) Phase 1 (stockperson standing stationary) and (**b**) Phase 2 (stockperson sitting stationary) of the home-pen test. Treatments: Control group (CG), dominant demonstrator group (DDG) and subordinate demonstrator group (SDG).

For all behavioral measures, the values and the effects of treatment are summarized in Table 5. During Phase 1, when the stockperson entered the home pen and remained standing, no effect of treatment on the latency to first physical contact (Kaplan–Meier, X^2^ = 0.89, *p* = 0.64, df = 2; Figure 3a) and the time of contact of pigs (F2/11.51 = 0.057, *p* = 0.94) were found. On average, pigs took 14.02 s (±2.42) to contact the stockperson and spent 69.48% (±4.08) of time in contact with the stockperson. However, differences were found between treatments regarding the frequency of contact (F2/11.84 = 5.509; *p* = 0.020). Pigs from CG showed a greater frequency of contact than SDG (*p* = 0.014) and DDG pigs (*p* = 0.018), whereas the DDG and SDG pigs did not differ from each other (*p* = 0.901). Regarding the time that pigs spent looking at the stockperson, an effect of treatment was found (Kruskal–Wallis, X^2^ = 7.608, *p* = 0.022, df = 2). Pigs from DDG and CG spent more time looking at the stockperson than the SDG pigs (*p* < 0.01), whereas the DDG and CG pigs did not differ from each other (*p* = 0.761). No significant effect of sex was found for the behavioral measures during Phase 1 (*p* > 0.10).

During Phase 2, while the stockperson was motionless sitting for 2 min, SDG pigs contacted the stockperson sooner (Kaplan–Meier, X^2^ = 18.83, *p* < 0.001, df = 2; Figure 3b) and spent more time in contact with the stockperson (Kruskal–Wallis, X^2^ = 13.53, *p* = 0.007, df = 2) than DDG and CG pigs, whereas DDG and CG did not differ from each other (*p* > 0.10). Furthermore, all pigs from SDG came into contact with the stockperson within 1 s, whereas 90% of pigs from DDG that approached made contact within 45 s (Figure 3b). No differences between treatments were found for the frequency of contact with the stockperson (F2/12.51 = 1.352; *p* = 0.294). Regarding the time that pigs spent looking at the stockperson, an effect of treatment was found (Kruskal–Wallis, X^2^ = 13.539, *p* = 0.001, df = 2). Pigs from DDG and CG spent more time looking at the stockperson than SDG pigs (*p* < 0.01), whereas DDG and CG pigs did not differ from each other (*p* > 0.10). There were no differences between treatments on frequency of climbing on the stockperson (F2/11.81 = 0.381, *p* = 0.691), or on the number of pigs climbing on the stockperson (CG: *n* = 13, DDG: *n* = 7, SDG: *n* = 9, Pearson’s chi-square = 1.301, *p* = 0.52, df = 2). No significant effect of sex was found for the behavioral measures during Phase 2 (*p* > 0.10).

During Phase 3, when the stockperson approached the pigs and tried to stroke them, significant differences were found between treatments for the percentage of strokes accepted (Kruskal–Wallis, X^2^ = 11.50, *p* = 0.003, df = 2) and the number of attempts required by the stockperson to complete the first stroke (Kruskal–Wallis, X^2^ = 12.78, *p* = 0.002, df = 2). Pigs from SDG accepted a higher percentage of strokes than DDG (*p* = 0.005) and CG pigs (*p* = 0.018), whereas DDG and CG did not differ from each other (*p* = 0.831). Regarding the number of attempts until accepting the first stroke, CG pigs needed more attempts than SDG pigs (*p* = 0.002) and tended to need more attempts until the first stroke than DDG pigs (*p* = 0.065). No differences were found between SDG and DDG pigs (*p* = 0.855). Moreover, 48% of animals from the control group accepted being stroked at the first attempt, while 79% and 95% of pigs from the DDG and SDG accepted being stroked at the first attempt, respectively. There were no significant differences between treatments on frequency of climbing on the stockperson (F2/13.74 = 0.279, *p* = 0.761) or the number of pigs climbing on the stockperson (CG: *n* = 14, DDG: *n* = 10, SDG: *n* = 11, Pearson’s chi-square = 0.176, *p* = 0.916, df = 2). However, there was a significant effect of sex for frequency of climbing (F1/57.45 = 4.769, *p* = 0.033), in which females (2.10 ± 3.93) exhibited a greater frequency than male pigs (1.08 ± 0.41).

## 4. Discussion

The present study examined whether demonstrator pigs from different social ranks receive different social attentiveness from their conspecifics during GH demonstrations. Our results indicate that individuals preferentially attended to dominant demonstrators rather than subordinates while the stockperson tried to provide them gentle handling. Additionally, we investigated the phenomenon of social facilitation and inhibition in the context of social learning related to a positive human–pig relationship. Specifically, we examined whether the presence of an experienced conspecific (demonstrator) of different social rank affects the motivation and behavior of their pen mates to positively interact with their stockperson. Our previous findings, obtained from the same individuals used in the present study, indicated that pigs are capable of perceiving their stockperson positively through observational social learning when they are individually tested [6]. However, the social context where the animals are confronted by the stockperson is critical in determining whether animals are able to demonstrate their socially acquired learning. This study shows that the expression of social learning in pigs regarding the positive perception of humans depends on social context and demonstrator identity during human interaction in a familiar social environment. In our study, the presence of a socially dominant demonstrator interfered with the motivation of their pen mates to positively interact with the stockperson, whereas the presence of a subordinate demonstrator facilitated the expression of a socially learned affinity toward the stockperson.

### 4.1. Behavioral Reaction of Observer Pigs during and after the Gentle Handling Sessions

The directed social learning theory indicates that information that can be acquired socially by an individual depends not only on dynamics and social context, but also on the demonstrator identity [40]. In this study, we focused on the demonstrator’s social identity to determine which social rank would be preferentially observed when a physical (but not visual) separation between the demonstrator and the observer pigs is incorporated during the GH sessions. This type of design facilitated quantification of how often and for how long individuals watched a demonstrator receiving gentle handling, without the potential physical inhibitory effect of the demonstrator’s social rank (e.g., physical displacements or direct aggression) [19].

Our results provide evidence that the social status of an experienced pig (demonstrator) mediates social attention received during the demonstrator’s interaction with the human. The way in which domestic pigs visually attended to demonstrator conspecific varied as a function of their social rank. During the GH demonstration sessions, there was an overall preference for observer pigs to visually attend to the dominant demonstrators more than the low-ranking demonstrators, which was consistent with other studies. The bias to preferentially attend to dominant over subordinate individuals has been widely documented in several primate species (chimpanzees, [18]; vervet monkeys, [41]; brown capuchin monkeys, [25]), including humans [42], while evidence in domestic animals is still scarce [19]. In this regard, Nicol and Pope [19] in a study with a design similar to ours, found that during the demonstration sessions, the number of laying hens per flock that watched dominant demonstrators performing a keypeck response to obtain food −behind a Perspex panel− was significantly higher than the number of hens who watched a subordinate demonstrator. According to our results, the rank of the demonstrator was an important factor in determining social attention in observer pigs. These findings have implications for extending our understanding of pigs’ cognition and social behavior, as well as the nature of social attention bias in farm animals.

To the best of our knowledge, this is the first study showing a social attention bias to high-ranking individuals in domestic pigs. However, it is important to highlight that this selective attention toward dominant demonstrators was notably accentuated during the second week of GH. In our study, observer pigs exposed to dominant demonstrators spent more time observing the DSI on the fifth day of the first week of GH, and every day of the second week, compared to pigs exposed to subordinates. In contrast, no differences were found between treatments during the first days of GH, observing a longer attentiveness to the demonstrator´s behavior during the first two days, regardless of their social rank. According to Raoult and Gygax [43], attention oriented to a stimulus can indicate either a vigilance state (e.g., toward a threat) or curiosity (e.g., for novelty). In several species, including pigs, it has been reported that threatening stimuli, as well negative emotional stimuli perceived in conspecifics, receive greater attention, and are processed preferentially [44,45,46]. For instance, Goumon and Špinka [45] showed that pigs increased visual attention toward a stressed conspecific compared to a non-stressed conspecific. Specifically, they found that pigs observing (through wire mesh partition) a pen mate in distress (exposed to restraint) spent more time in close proximity to partition looking at their pen mate. In our study, the greater attention and proximity to panel observed in all pigs during the first two days of the demonstrator´s handling might be explained by the aversive nature of initial handling. It is possible that the observer pigs perceived the initial DSI (capturing and isolating the demonstrator pig for the GH session) to be a threatening stimulus. However, measurements of autonomic nervous system activity, such as heart rate and heart rate variability could have been useful physiological measures to support these findings.

While this study demonstrates the relevance of a threatening stimulus for attracting visual attention of observer pigs, an open question is to what extent this attention is influenced by the emotional valence (negative or positive) of social information. Regarding why the animals paid significantly greater attention to dominant rather than subordinate demonstrators from the fifth day of the first week through the end of the second week, we hypothesize that it might be due to the positive valence of the situation experienced by the demonstrator. It is likely that the demonstrators perceived the GH sessions positively in the second week, which resulted in a greater acceptance of stroking and sucrose solution. Although we did not evaluate the behavior of the demonstrator toward the stockperson, a study in miniature pigs performed by Tanida and Nagano [47] showed that after five days of gentle handling (strokes, soft voice, and reward food) the pigs quickly initiated physical contact with the handler, maintaining this speed of approach over time. This study suggests that pigs may become habituated to humans after one week of GH, thus supporting our hypothesis. On the other hand, research on visual attention has shown that negative stimuli receive greater attention and have a greater impact on information processing than positive stimuli when they are both presented simultaneously [48,49]. However, when enough attention resources are available, for instance, when stimuli are not presented simultaneously, as was observed in our study, positive information can be processed in the same way as negative information. Thus, negativity bias does not necessarily present itself in circumstances of sufficient attention [50]. This attentional mechanism might be useful to explain why the pigs in the present study showed significantly greater attention during the first two days (negative information), and from the fifth day of the first week through the end of the second week (positive information).

When the time that pigs spent in physical contact with the panel was analyzed, we found that observer pigs exposed to dominant demonstrators spent more time in proximity on the fourth and fifth day of the first week and the first three days of the second week. The proximity with the panel could reflect a greater motivation of the pigs, either for observing the behavior of the demonstrator and/or to try to re-establish physical contact with her/him through the panel’s holes. Social attention studies in which an animal is presented with the opportunity to watch an individual through a peephole have been previously performed in several species (marmosets, [51]; brown Capuchin Monkeys, [25]; keas, humans, and dogs, [52]). In those studies, if the animals were highly motivated to watch the behavior of their conspecific (located behind an opaque panel), they would approach the hole to observe that individual on the other side of the panel. This type of experimental design is useful in assessing the motivation of animals to observe another individual [52]. In our study, pigs were allowed to watch the demonstrator through a transparent panel with some holes, which allowed partial snout-to-snout contact between animals. Therefore, our design does not allow us to differentiate whether the motivation to remain close to the panel was just to observe the demonstrator’s behavior or attempt to physically interact with her/him.

Alternatively, a search for contact with the dominant demonstrator through the panel’s holes could have been motivated by an attempt to reduce their stress due to social separation (social support). This is supported by Goumon and Špinka [45], who demonstrated empathic responses in pigs (e.g., snout contact, proximity, head orientation) through a wire mesh partition toward restrained conspecifics. Another possible explanation might be to obtain information on the demonstrator’s current situation. It is known that pigs can transfer and acquire information (such as new food preferences) by brief snout–snout interactions with experienced conspecifics [53]. Therefore, one possibility might be that pigs were attracted to contact the acrylic panel in order to collect information present in the oral cavity of the demonstrator after the sucrose solution consumption. However, our data obtained immediately after the animals were re-grouped do not appear to fully support this theory. When the demonstrators had returned to their social group, no differences between the treatment groups were found regarding the time and frequency of snout–snout contact between observers and demonstrators. Instead, we observed very little interaction between the animals.

### 4.2. Behavior of the Observer Pigs toward the Stockperson in the Home-Pen Test

When the stockperson entered the home pen and remained standing and motionless, no differences between treatments were found in pigs’ motivation to approach and contact the stockperson. Previous studies have shown that pigs are highly sensitive to the body posture adopted by humans [9]. More precisely, they take more time to approach a person when he or she remains erect compared to a person sitting or squatting [54,55]. This could explain why the animals, regardless of the treatment, did not immediately approach the stockperson. Nevertheless, when the stockperson adopted a sitting posture, the observer pigs exposed to subordinate demonstrators expressed a higher attraction to the stockperson than the other treatment groups. The attraction to the stockperson was characterized by the latency to approach her, the time spent in contact with her, and the acceptance of strokes. Pigs exposed to subordinate demonstrators immediately approached and spent almost the entire time exploring the stockperson. Additionally, they exhibited a reduced avoidance response toward the stockperson when she attempted to stroke them, which resulted in a higher percentage of accepted strokes, indicating a clear positive perception of the human [5,7,56]. Taken together, these results suggest that observer pigs’ motivation to positively interact with the human was intensified or at least enabled by the presence and behavior of a low-ranking demonstrator conspecific during the GH sessions. Previous social learning studies proposed social facilitation as the responsible mechanism when animals show an increased motivation to engage in the same behaviors observed in a conspecific [57]. In the present study, all demonstrators naturally showed a high affinity toward the stockperson. However, interest in interacting with the human was significantly accentuated in those pigs who were in presence of socially subordinate demonstrators. Therefore, positive handling of previously selected subordinate demonstrators seems to be the best strategy to reduce the level of fear in large groups of pigs.

On the other hand, animals exposed to dominant demonstrators were more cautious with the stockperson during the home-pen test, behaving similarly to the control pigs. Overall, both treatment groups (DDG and CG) expressed lower motivation to interact with the stockperson, as indicated by their longer times to approach her, the lower percentage of strokes accepted, and the longer time they spent looking at the stockperson in an attentive attitude. The attentive attitude, i.e., head oriented toward a stimulus, is often displayed in response to a novel or aversive stimuli in order to gather information about the current situation [5,8]. A greater time gazing at a stimulus has been associated with an increased vigilance state as a sign of fear [58]. Unlike the observer pigs, control pigs were not familiarized with the presence of their stockperson inside the pen. Therefore, it is expected that the presence of the stockperson motionless in the home pen attracted greater attention by control pigs [8], either by curiosity or because she was perceived as a threatening stimulus [5]. Alternatively, the minimal contact with the stockperson throughout the experimental period could explain the greater latency to physical contact and the low percentage of accepted strokes in control pigs. These behaviors, commonly associated with greater levels of fear toward humans, have been previously reported in pigs receiving minimal human contact, as shown by de Oliveira et al. [59] and Tallet et al. [5]. However, physiological measurements, such as heart rate variability [6,60] or cortisol concentrations [61], would have been necessary to conclude whether fear was the predominant emotional state in these animals in any of the phases.

Regarding the behavior shown by observer pigs who were in presence of socially dominant demonstrators, our results show that the presence of high-ranking demonstrators decreased their motivation levels to positively interact with the human. These results contrast with those reported by Luna et al. [6], who, evaluating these same subjects outside their social group, found that observer pigs showed high motivation to positively interact with the stockperson regardless of whether they were previously exposed to dominant or subordinate demonstrators. In social isolation, all the observer pigs positively perceived the human as a result of observational social learning [6]. The “failure to perform” hypothesis described by Drea and Wallen [31] may offer one possible explanation for the discrepancy of the results. According to this hypothesis, there are no differences in cognitive skills of learning between individuals of different social rank. This means that lower-ranking individuals can learn in all social contexts. However, they are unsuccessful in expressing their learning (or knowledge), either intentionally or due to social inhibition exerted by the presence of a dominant animal, as was observed in the present study. Thus, according to the “failure to perform” hypothesis, it is expected that observer pigs exposed to dominant demonstrators who exhibited high affinity to the human in isolation show, subsequently, a decline in their affinity when a high-ranking demonstrator is present in the group. Consistent with our findings, several studies in nonhuman primates have documented that low-ranking animals more effectively expressed their learning performance when segregated from higher-ranking animals [30,31,62]. Thus, our results add to the evidence demonstrating the effect of social dynamics within the group on the facilitation and inhibition of socially acquired learning in domestic animals.

Social inhibition by dominant individuals could be assumed to occur for strategic reasons. Dominant individuals are central in the social network [63], which can result in a social attentional bias toward these individuals [18,19]. Additionally, they have been shown to be more successful in domains such as foraging and reproduction [64], which gives them priority to access resources [65]. It has also been shown that dominant individuals are more likely to initiate agonistic interactions in defense of a valuable and limited resource [30,66] and are better able to limit the subordinates’ access to preferred resources [27]. In our study, the human could be considered as a positive conditioned stimulus that announces the arrival of positive reinforcement [9]. This means that the stockperson, by providing strokes and a palatable solution, could be perceived as a source of rewarding experiences for the demonstrator pigs, thus becoming a potentially defendable and monopolizable resource by the dominant demonstrators. This could explain the longer time that observer pigs stayed away from the stockperson in an attention attitude directed to her, possibly to evade competitive situations that might result in intimidation or aggression from the dominant demonstrators [64]. However, it is important to note that we did not observe obvious signs of aggression or threat directed from the dominant demonstrators toward their pen mates in order to disrupt the interaction with the stockperson, demonstrating that social constraints can occur in the absence of direct aggression, as previous studies have shown [31,62]. According to Drea and Wallen [31], it is possible that subtle cues emitted by dominant demonstrators, and not easily perceived by the experimenter, affect the behavior of their low-ranking groupmates. Pigs, unlike other species, show reduced agonistic behavior after the establishment of the social hierarchy, preferring distance threats, exchanges of glances, or active avoidance behavior to maintain rank order [67,68]. Therefore, given this level of social interaction and organization, it is expected that observer pigs exposed to the dominant demonstrators have spent more time looking at the stockperson in an attentive attitude and took longer to approach her.

On the other hand, it is known that pigs have cognitive abilities that allow them to identify and recognize individual humans [9], as well as remember [69] and anticipate events [70]. Assuming such abilities, added to the fact that lower-ranking pigs maintain their distance from dominant individuals when confronted with a food resource [71], it is possible that lower-ranked observers may have anticipated the response of the dominant demonstrators toward the stockperson. This could have caused the observers to remain in a vigilant state for a longer time while monitoring the stockperson and waiting for the demonstrators to establish physical contact with the human and reward (i.e., sucrose solution).

## 5. Conclusions

This study is the first to clearly demonstrate preferential social attention to high-ranking demonstrator pigs, rather than those of low rank, especially when the demonstrator seems to experience a situation of positive valence. On the other hand, when observer pigs were evaluated in a familiar social environment accompanied by the demonstrator and their pen mates, we found that pigs’ reaction toward the stockperson was modulated by the presence and social rank of the demonstrator conspecific. Specifically, observer pigs were more cautious when interacting with the stockperson while a socially dominant demonstrator was present in the home pen. Further insight into the nature of the social dynamics that influenced the expression (or inhibition) of learning can be obtained by comparing the results obtained here with those reported in the previous study, where the same subjects were tested individually [6]. Taking results from both experiments into account, it is possible to conclude that manipulation of the social context in which the learning is evaluated (i.e., individual or group testing) can lead to a markedly different view of the pigs’ cognitive ability to express a socially learned affinity toward humans. The observer pigs’ decline in motivation to interact with the stockperson as a result of the presence of dominant demonstrators shows that learning in pigs should be evaluated in a context free of social constraints. Finally, taken together, these findings highlight the need to understand how farm animals are socially influenced by the dynamics, the social context, and the social identity of group members in order to design effective strategies that reduce socially induced welfare issues.

## Figures and Tables

**Table 1:** Timeline of the experiment.

Day of the Experiment	Pigs Age (Days)	Event/Test	Place	Measurement
1	21	Weaning	Commercial farm	-
1–7	21–27	Acclimation to nursery pens	Home pen	-
1–2	21–22	Agonistic behavior video collection	Home pen	Behavioral
3–5	23–25	Agonistic behavior video analysis	-	Behavioral
6	26	Dominance Index estimation	-	Dominance index
8–40	28–60	Gentle handling (GH) sessions	Home pen	Behavioral
8–12	28–32	Behavioral video collection during and after GH sessions	Home pen	Behavioral
15–19	35–39	Behavioral video collection during and after GH sessions	Home pen	Behavioral
41–42	61–62	Home-pen test	Home pen	Behavioral

**Table 2:** Behavioral observations evaluated during and after the gentle handling (GH) sessions that occurred between 9:00 and 11:00 a.m. (Monday–Friday) on experimental days 8–12 and 15–19.

Behavioral Observations during GH Sessions	Description
Looking at the demonstrator-stockperson interaction	Time (%) that the observer pig stood still within 20 cm of the acrylic panel with its head oriented toward the demonstrator–stockperson dyad while the demonstrator was gently handled by the stockperson. The percentage of time was calculated as a function of the entire 10 min during the GH session.
Frequency of looking at the demonstrator–stockperson interaction	Number of times the observer pig stood still within 20 cm of the acrylic panel with its head oriented toward the demonstrator–stockperson dyad while the demonstrator was gently handled by the stockperson.
Time of contact with the panel	Time (%) the observer pig spent with their snout physically contacting the acrylic panel and/or the holes of the panel (touching or sniffing) with their head oriented toward the demonstrator–stockperson dyad. The percentage of time was calculated as a function of the entire 10 min during the GH session.
Frequency of contact with the panel	Number of times the observer pig physically contacted the acrylic panel with their snout, while their head was oriented toward the demonstrator-stockperson dyad.
Frequency of climbing on the panel	Number of times the observer pig’s two front legs made contact with the acrylic panel in a single motion while standing on its two hind legs.
**Behavioral Observations after GH Sessions**	
Time of snout–snout contact	Time (%) the observer pig spent touching the end of the demonstrator’s snout with the end of its own snout. The percentage of time was calculated as a function of the entire 3 min after the GH session.
Frequency of snout–snout contact	Number of times the observer pig touched the demonstrator pig’s snout with its own snout.

% = percentage.

**Table 3:** Behavioral observations evaluated during the interaction with the stockperson in each phase of the home-pen test.

Behavioral Observation	Description	Phase
Latency to first physical contact	Time (s) taken for the pig to make physical contact with any part of the stockperson’s body.	1,2
Time of physical contact	Time (%) pig stayed in physical contact with any part of the stockperson’s body, either touching or sniffing. The percentage of time was calculated as a function of the minutes elapsed for each phase.	1,2
Looking at the stockperson	Time (%) pig stood still looking at the stockperson, with its body and head oriented toward the stockperson without physically interacting with her. The percentage of time was calculated as a function of the minutes elapsed for each phase.	1,2
Frequency of contact	Number of times the pig physically contacted any part of body of the stockperson.	1,2
Frequency of climbing on the stockperson	Number of times the pig was observed climbing on the stockperson, with at least the front legs on the thighs.	2,3
Animals climbing on the stockperson	Number of animals that were observed climbing on the stockperson, with at least the front legs on the thighs.	2,3
Accepted strokes	Strokes (%) accepted by each pig based on the total attempts made by the stockperson. The percentage of strokes was calculated as a function of the total attempts made by the stockperson during Phase 3.	3
Attempts required until accepting the first stroke	Number of attempts made by the stockperson until the pig accepted the first stroke.	3

s = second; % = percentage.

**Table 4:** Observer pig behavioral responses during and after the demonstrator pig gentle handling sessions during week 1 and week 2, according to their treatment.

Behavior	Week	Treatments		*p*-Value
		DDG	SDG	
		(*n* = 20)	(*n* = 20)	
During GH sessions				
Looking at the DSI (%)	Week 1	43.98 ± 3.26 ^p^	35.65 ± 3.14 ^q^	0.077 ^1^
	Week 2	43.09 ± 2.22 ^a^	20.52 ± 2.14 ^b^	<0.001 ^1^
Frequency of looking at the DSI	Week 1	9.35 ± 0.54	9.05 ± 0.52	0.678 ^2^
	Week 2	8.44 ± 0.57 ^a^	6.48 ± 0.55 ^b^	0.019 ^2^
Time of contact with the panel (%)	Week 1	35.97 ± 3.03 ^p^	27.81 ± 2.92 ^q^	0.071 ^1^
	Week 2	32.67 ± 2.24 ^a^	17.99 ± 2.16 ^b^	<0.001 ^1^
Frequency of contact with the panel	Week 1	12.61 ± 0.84	11.78 ± 0.81	0.484 ^2^
	Week 2	9.97 ± 0.74 ^a^	7.70 ± 0.71 ^b^	0.035 ^2^
Frequency of climbing on the panel	Week 1	0.45 ± 0.14	0.32 ± 0.14	0.473 ^3^
	Week 2	0.44 ± 0.10	0.44 ± 0.10	0.961 ^3^
After GH sessions				
Time of snout–snout contact (%)	Week 1	1.30 ± 0.24	0.84 ± 0.23	0.170 ^1^
	Week 2	0.99 ± 0.27	0.88 ± 0.26	0.759 ^1^
Frequency of snout–snout contact	Week 1	1.02 ± 0.05	0.95 ± 0.05	0.319 ^3^
	Week 2	0.47 ± 0.10	0.49 ± 0.10	0.874 ^3^

Values within a row with different letters significantly differ (^a,b^: *p* < 0.05) or tended to differ (^p,q^: 0.05 < *p* < 0.1). DSI = demonstrator–stockperson interaction; DDG = dominant demonstrator group; SDG = subordinate demonstrator group. ^1^ mixed ANOVA based on angular transformation (df = 1); ^2^ mixed ANOVA model (df = 1); ^3^ mixed ANOVA model based on square root transformation (df = 1). Data analyzed with mixed ANOVA models are expressed as estimated marginal means and standard error means (E.M.M. ± S.E.M.) of non-transformed data.

**Table 5:** Pig behavioral responses during the different phases of the home-pen test, according to their treatment.

Behavior		Treatments		*p*-Value
	CG	DDG	SDG	
	(*n* = 25)	(*n* = 20)	(*n* = 20)	
Phase 1				
Latency to first contact (s)	10.41 ± 2.34	16.67 ± 5.04	15.88 ± 5.19	0.640 ^2^
Time of contact (%)	69.70 ± 10.21	70.95 ± 10.59	67 ± 10.60	0.940 ^1a^
Frequency of contact	2.40 ± 0.25 ^a^	1.35 ± 0.27 ^b^	1.30 ± 0.27 ^b^	0.020 ^1^
Time looking at the stockperson (%)	10.67 (3.83–22.49) ^a^	6.15 (0–16.94) ^a^	1.04 (0–9.28) ^b^	0.022 ^3^
Phase 2				
Latency to first contact (s)	3.80 ± 0.82 ^a^	16.29 ± 8.03 ^a^	1.00 ± 0 ^b^	<0.001 ^2^
Time of contact (%)	86.67 (72.86–88.44) ^a^	84.88 (81.54–88.27) ^a^	100 (96.53–100) ^b^	0.007 ^3^
Frequency of contact	3.44 ± 0.52	2.20 ± 0.54	2.70 ± 0.54	0.294 ^1^
Time looking at the stockperson (%)	10.04 (0–16.40) ^a^	10.75 (0–15.12) ^a^	0 (0–0) ^b^	0.001 ^3^
Climbing on the stockperson (frequency)	1.44 ± 0.52	0.80 ± 0.53	1.25 ± 0.53	0.691 ^1^
Phase 3				
Accepted strokes (%)	76.47 (27.14–100) ^a^	82.35 (62.50–93.33) ^a^	100 (93.68–100) ^b^	0.003 ^3^
Attempts until accepting the first stroke	2.00 (1.00–4.50) ^a^	1.00 (1.00–1.00) ^a,b^	1.00 (1.00–1.00) ^b^	0.002 ^3^
Climbing on the stockperson (frequency)	1.88 ± 0.48	1.57 ± 0.53	1.35 ± 0.52	0.761 ^1^

Values within a row with different letters significantly differ (^a,b^: *p* < 0.05). CG = control group; DDG = dominant demonstrator group; SDG = subordinate demonstrator group. Phase 1 = stockperson standing stationary; Phase 2 = stockperson sitting stationary; Phase 3 = stockperson moving. ^1^ mixed ANOVA model; ^1a^ mixed ANOVA based on angular transformation (df=2); ^2^ Kaplan–Meier analysis (df = 2); ^3^ Kruskal–Wallis test (df = 2). Data analyzed with mixed ANOVA models are expressed as estimated marginal means and standard error means (E.M.M. ± S.E.M.) of non-transformed data. Data analyzed with the Kaplan–Meier method are expressed as means and standard error (m ± S.E.) and data analyzed with Kruskal–Wallis test are expressed with medians and interquartile ranges (md (Q25–Q75)).

## Data Availability

The data presented in this study are available from the corresponding author upon reasonable request.
